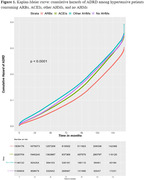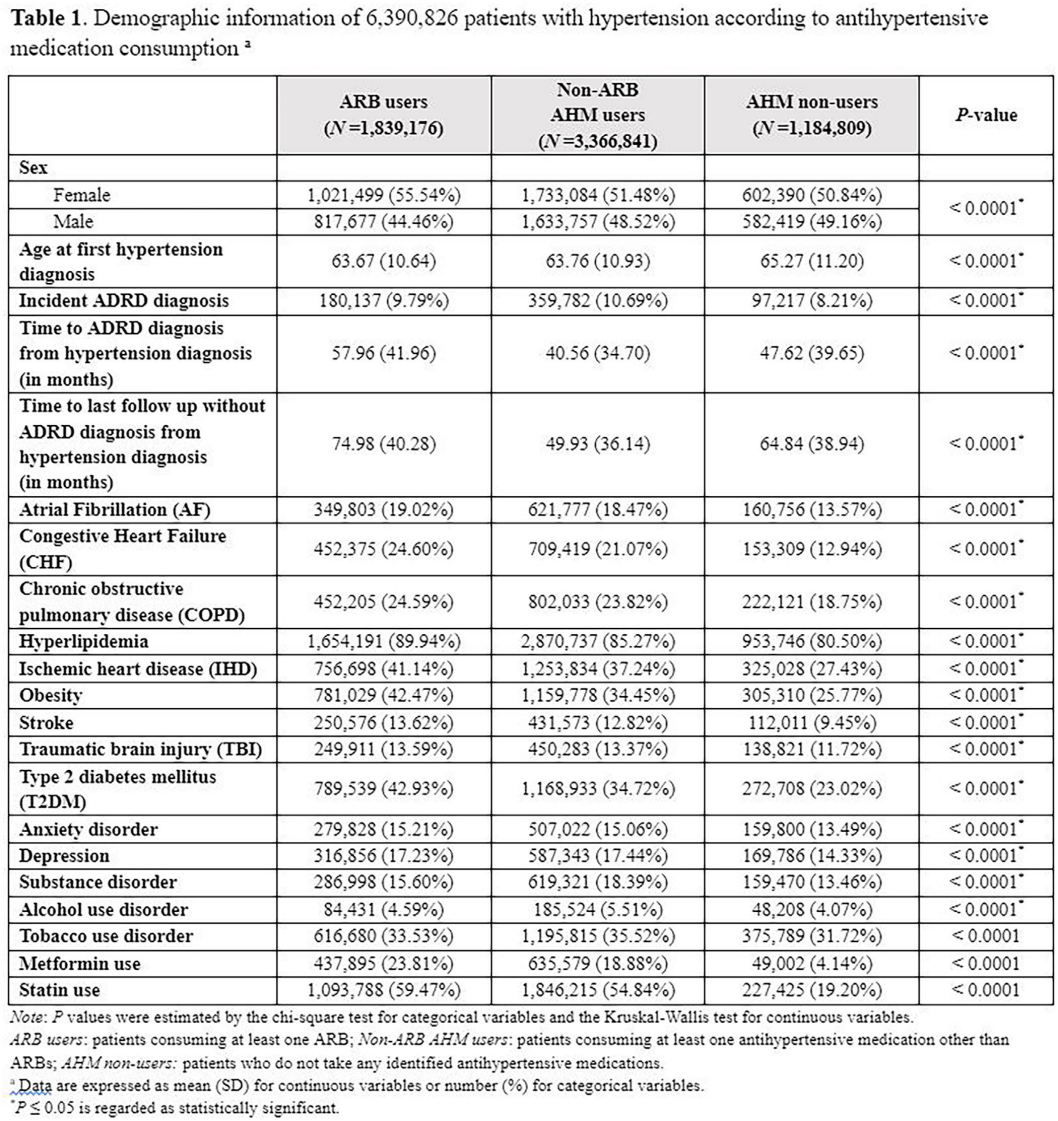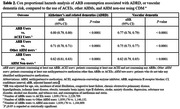# Lower Risk of Alzheimer’s Disease and Related Dementias associated with Angiotensin Receptor II Blockers (ARBs) treatment for individuals with Hypertension in High‐volume Claims Data

**DOI:** 10.1002/alz.084745

**Published:** 2025-01-09

**Authors:** Sori Kim Lundin, Xinyue Hu, Jingna Feng, Karl Kristian Lundin, Lu Li, Yong Chen, Paul E Schulz, Cui Tao

**Affiliations:** ^1^ Center for Biomedical Semantics and Data Intelligence (CBSDI), University of Texas Health Science Center at Houston, Houston, TX USA; ^2^ School of Public Health, The University of Texas Health Science Center at Houston, Houston, TX USA; ^3^ School of Biomedical Informatics, The University of Texas Health Science Center at Houston, Houston, TX USA; ^4^ Baylor College of Medicine, Houston, TX USA; ^5^ Perelman School of Medicine, University of Pennsylvania, Philadelphia, PA USA; ^6^ John P. and Kathrine G. McGovern Medical School at UTHealth, Houston, TX USA; ^7^ McWilliams School of Biomedical Informatics, The University of Texas Health Science Center at Houston, Houston, TX USA

## Abstract

**Background:**

Findings regarding the protective effect of Angiotensin II receptor blockers (ARBs) against Alzheimer’s disease and related dementias (ADRD) and cognitive decline have been inconclusive.

**Method:**

A total of 6,390,826 hypertensive individuals were included in this study from Optum’s de‐identified Clinformatics® Data Mart. We identified antihypertensive medication (AHM) drug classes and subclassified ARBs by blood‐brain barrier (BBB) permeability. We compared baseline characteristics and used the Kaplan‐Meier (KM) survival curve and adjusted Cox proportional hazards (PH) model for survival analyses.

**Result:**

AHM non‐users (*N* = 1,184,809) had a lower incidence of all comorbidities and consumption of metformin and statins compared to ARBs users (*N* = 1,839,176) and non‐ARBs AHM users (*N* = 3,366,841) (all *P*<0.0001). The KM curve showed that ARB users had lower cumulative hazard than other AHM users or AHM non‐users (*P*<0.0001). In Cox PH analysis, ARB users showed a 20% lower adjusted hazard of developing ADRD compared to angiotensin‐converting enzyme inhibitor (ACEI) users and a 29% and 18% reduced risk when compared to non‐ARB/ACEI AHM users and AHM non‐users (all *P*<0.0001). Consumption of BBB‐crossing ARBs was linked to a lower risk of ADRD development than non‐BBB‐crossing ARBs, undetermined ARBs, and non‐consumption of AHMs by 11%, 25%, and 31% (all *P*<0.0001).

**Conclusion:**

This study suggests that ARBs are superior to ACEIs, non‐ARB/ACEI AHMs, or non‐use of AHMs in reducing the risk of ADRD among hypertensive patients. Also, BBB‐permeability in ARBs was associated with lower ADRD risk. There is no cure for AD, ADRD, or vascular dementia; hence, these findings are significant in preventing those disorders in an inexpensive, convenient, and safe way.